# Prion-like Domains in Eukaryotic Viruses

**DOI:** 10.1038/s41598-018-27256-w

**Published:** 2018-06-12

**Authors:** George Tetz, Victor Tetz

**Affiliations:** Human Microbiology Institute, New York, NY 10027 USA

## Abstract

Prions are proteins that can self-propagate, leading to the misfolding of proteins. In addition to the previously demonstrated pathogenic roles of prions during the development of different mammalian diseases, including neurodegenerative diseases, they have recently been shown to represent an important functional component in many prokaryotic and eukaryotic organisms and bacteriophages, confirming the previously unexplored important regulatory and functional roles. However, an in-depth analysis of these domains in eukaryotic viruses has not been performed. Here, we examined the presence of prion-like proteins in eukaryotic viruses that play a primary role in different ecosystems and that are associated with emerging diseases in humans. We identified relevant functional associations in different viral processes and regularities in their presence at different taxonomic levels. Using the prion-like amino-acid composition computational algorithm, we detected 2679 unique putative prion-like domains within 2,742,160 publicly available viral protein sequences. Our findings indicate that viral prion-like proteins can be found in different viruses of insects, plants, mammals, and humans. The analysis performed here demonstrated common patterns in the distribution of prion-like domains across viral orders and families, and revealed probable functional associations with different steps of viral replication and interaction with host cells. These data allow the identification of the viral prion-like proteins as potential novel regulators of viral infections.

## Introduction

Recently, prions and their infectious forms have attracted a lot of research attention^1,2^. The infectious prion forms (PrPSc) represent the misfolded normal proteins (PrPC) and were shown to be infectious, since then can self-propagate and interact with the endogenous PrPC, catalyzing their conversion into pathological PrPSc^3–7^. Previously they had been primarily known as the inducers of transmissible spongiform encephalopathies, however, today they have been shown to be involved in the development of a variety of neurodegenerative diseases^8–10^.

Recently, the abnormal conformation of self-propagating PrPScs was found to be associated with the formation of the toxic, misfolded, insoluble, and highly-ordered fibrillar cross-β aggregates of β-amyloid, *tau*, and TDP-43 proteins in Alzheimer’s disease and amyotrophic lateral sclerosis^11–14^. The follow-up studies demonstrated that the pathological protein conversion and the deposition of the insoluble protein aggregates are associated with the development of other diseases, including Parkinson’s, Huntington, fatal familial insomnia, ataxias, diabetes, and others^9,15,16^.

However, protein misfolding was shown to play important physiological roles as well in eukaryotes and prokaryotes^17–21^. Self-perpetuating properties of prions are important for the formation of bacterial and fungal biofilms, bacterial bacteriocin functioning, molecular transport and secretion, and the preservation of long-term memory in yeasts^22–25^. Moreover, prions were recently shown to participate in the communication between prokaryotes and eukaryotes, resulting in the alterations in *Caenorhabditis elegans* amyloid formation following its colonization with amyloid-producing *Escherichia coli*^26^.

Although the molecular mechanisms underlying *de novo* prion formation remains elusive, the aggregation of PrPs is an amino-acid sequence-dependent process. Most prions contain specific domains enriched in asparagine (Q) and glutamine (N), which, together with the average residue hydrophobicity and net sequence charge, allowed the development of algorithms for the identification of candidate prionogenic domains (PrDs) based on the hidden Markov model (HMM)^20,27–30^.

The HMM is currently used in many bioinformatic approaches for the statistical representation of prion domains, which allow, using the probabilistic sequence model of maximum likelihood estimation, to evaluate the compositional similarity of proteins and prions. One of these approaches is prion-like amino acid composition (PLAAC) analysis, which allows the evaluation of proteins containing PrDs, defined as domains with the compositional similarity to yeast prion domains, based on amino-acid interactions^27,31^. The resulting log-likelihood ratio (LLR) indicates the possibility that the analyzed protein is a prion. Using PLAAC algorithms, PrDs defined as domains shown to contain at least a domain compositionally similar to yeast prions, have recently been investigated in different eukaryotic and prokaryotic species, confirming their important regulatory and functional roles^20,32–34^. There are other algorithms, such as PAPA and PrionW, using an experimentally derived prion propensity score combined with explicit consideration of the intrinsic disorder, that help to predict prion domains bioinformatically^35–38^. Recently, we investigated the PrDs in phagobiota and determined that these domains can be found in bacterial and archaeal virus families, which increased our understanding of their possible interplay with microbiota and implication for human health^39^.

Similar to the bacterial viruses, eukaryotic viruses are found in nearly all ecosystems and they infect different types of organisms, including animals, insects, protists, and plants, but their life cycle is similar in different organisms, comprising the attachment, entry, biosynthesis of viral nucleic acids and proteins, maturation, and release of progeny^40^. The nature of the viral replication cycle leads to their pathogenicity, although certain viral species induce persistent infections^41^. Viruses are efficiently disseminated by horizontal and vertical transmission, and they are the causative agents of many devastating diseases, such as flu and some cancers^42,43^. However, the detailed molecular mechanisms underlying the pathological processes have not be completely elucidated yet^14,44^.

Moreover, despite previous efforts, the presence of PrDs in eukaryotic viruses has not been described very well and the PrDs have been identified in only several viral families^19,34^. Therefore, the PrDs distribution in different viral families and species and their functionalities have not been determined to date.

Here, we performed a detailed study of the putative prion domains in all known eukaryotic viruses. Using an HMM algorithm, we retrieved all available eukaryotic viral protein sequences from the UniProt KB database^44^. To the best of our knowledge, this is the most extensive effort aimed at the identification of candidate PrD sequences among eukaryotic viruses. Furthermore, we analyzed the regularities in the distribution of PrDs in different viral taxes, correlation of this distribution with viral structure, viral hosts, and protein functions. The PrDs were identified using different algorithms, including Gene Ontology (GO)^45^. Our results may contribute to the better understanding of the host-viral interactions and the relationship between viral prions and pathogenicity.

## Materials and Methods

### Protein sequences

To identify the PrDs present in viral proteomes, protein sequences were obtained from the UniProt KnowledgeBase (Swiss-Prot and TrEMBL). Protein functions were predicted using the GO terms and manually curated using the information from the UniProt database, the National Center for Biotechnology Information (NCBI), and the literature data^46^.

### Identification of PrDs in viral proteomes

The presence of PrDs in viral proteomes was analyzed in the known viruses, excluding bacteriophages, using the PLAAC prion prediction algorithm, based on the HMM, and the identification of PrDs was based on the compositional bias towards asparagine and glutamine aminoacyls, an average residue hydrophobicity, and the net charge of sequences. The output probabilities for the PrDs states in the PLAAC were constructed based on the amino-acid frequencies in the PrDs of *Saccharomyces cerevisiae*. Consequently, this basis can be altered, using the parameter “Alpha value”, which allows a continuous interpolation between organism-specific background frequencies (Alpha = 0.0) and *S. cerevisiae* background frequencies (Alpha = 1.0). Here, we used Alpha = 0.0, representing species-independent scanning, to identify the PrDs.

For the analysis, we have adjusted the total number of viral proteins contained in the UniProt database, since in the proteomes of different viruses, multiple fragments of the same proteins had multiple representation. Therefore, multiple copies of the same sequences were removed in Excel (Windows 10) using the ‘remove duplicates’ function. We used a low LLR cutoff of 0.003, in order to analyze the majority of PrDs and their distribution among different viral orders and families, and 2,681 PrDs have been identified (Supplementary Table 1). Prion-like domains of top 100 scoring PrDs in different viral species were also predicted by the program PAPA using default values and a defined cutoff score of 0.05 for prediction of the prion versus non-prion proteins (Supplementary Table 6)^35,36^. The regularities in the likelihood of the identified PrDs to be prions, and their distribution among different viral orders and families were analyzed. The functions of proteins with the identified PrDs were classified using the manually-curated GO categories and were based on the major steps of viral replication. A heatmap was generated using R-statistical computing (www.r-project.org) with the “levelplot” package. The values in the heatmap range between the lowest (blue) and the highest (red) LLR values.

### Statistical analysis

All statistical analyses were conducted using package Statistica for Windows (version 5.0) (StatSoft, Inc.). Data were compared between the viral orders, families, and species by using a χ^2^ test or the Fisher’s exact test. To detect differences in multiple comparisons, one-way analysis of variance (ANOVA) was fitted with the standard confidence interval of 95%. All results were considered statistically significant for p < 0.05.

## Results

### Identification of PrDs in viral proteomes

Using the prion-prediction PLAAC algorithm, we identified 2,679 PrDs in proteins from 735 different viruses. In total, we analyzed 2,742,160 proteins derived from the UniProtKB database from over 3000 known viral species (the whole virome discovered up-to-date) according to the International Committee on Taxonomy of Viruses (ICTV) (Supplementary Tables 1, 2)^47^.

**Figure 1:**
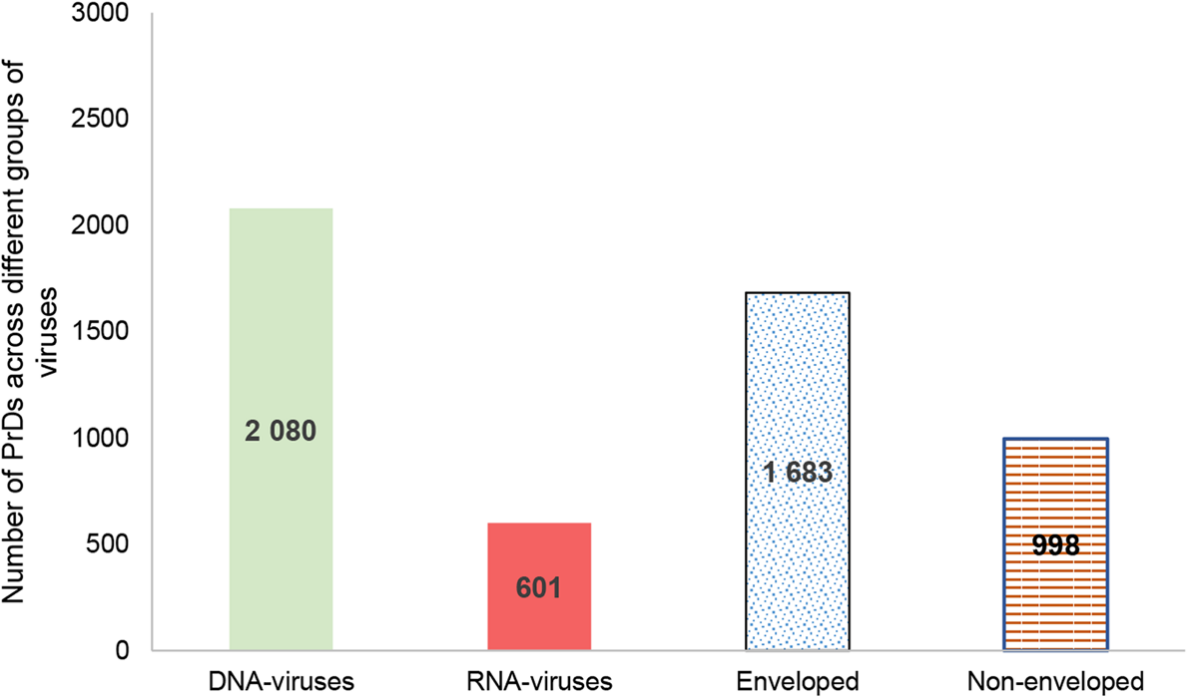
PrD enrichment in the proteome of different viruses. Numbers insider bars represent the total numbers of PrDs identified in each group.

The average numbers of LLRs varied between the DNA and RNA containing viruses as well as between the enveloped and non-enveloped ones. PrDs were more frequently found in the DNA-containing viruses. Enveloped viruses were also more frequently found to harbor PrD compared with the non-enveloped ones (Fig. 1).

**Figure 2:**
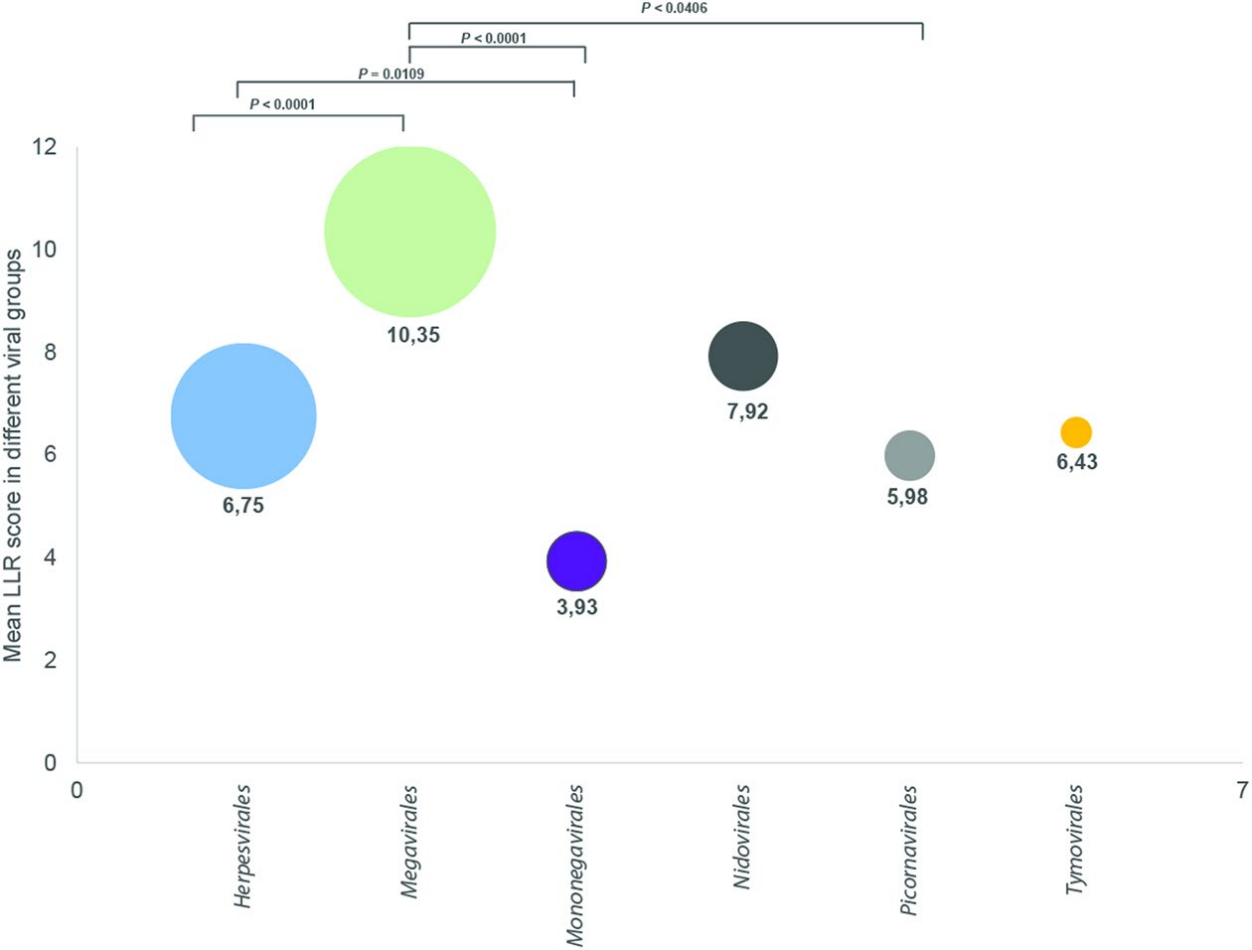
PrD enrichment in viral proteomes and the LLR scores. The ratio between PrD-containing proteins in each group and the total number of viral proteins is presented. Numerical values are medial LLR scores of the PrDs, and the circle size indicates the number of identified PrDs. Data were analyzed using one-way ANOVA.

High levels of PrDs were found in *Herpesvirales*, *Megavirales*, *Mononegavirales*, *Nidovirales*, *Picornavirales*, and *Tymovirales* (Fig. 2) (the members of the unassigned viral orders, represented by different unrelated families, are presented in Suppl. Table 2). The distribution of PrDs was shown to vary, with the highest prevalence found in *Herpesvirales* (LLC = 6.54).

To analyze the presence of PrDs in different viral orders, we evaluated the ratio between the species identified in this study to possess at least one PrD and the total number of different viral species within that order (Table 1)^47^.

**Table 1:** PrD enrichment in the proteomes of different viral orders.

Order	Number of PrD-containing species within one order	Total number of species within an order	PrD-containing species as the percentage of the total species number	P-value
*Herpesvirales*	74	103	71,84%	<0.0001
*Megavirales*	78	ND	ND	ND
*Mononegavirales*	35	212	16,51%	<0.0001
*Nidovirales*	60	64	93,75%	<0.0001
*Picornavirales*	44	138	31,88%	0.7579
*Tymovirales*	16	179	8,94%	0.948
Unassigned	427	2467	17,31%	<0.0001

We found that the highest number of PrD-containing species are found among *Nidovirales* and *Herpesvirales*, with over 93.75% and 71.84% of species, respectively, containing PrDs, while the lowest numbers were found in *Tymovirales*, with only 8.94% of species with identified PrDs. We have not included the results of *Megavirales* analysis due to the lack of classification data for this novel viral order^48^.

Furthermore, we calculated the mean number of PrDs per species, as the ratio of the total number of PrDs identified in viral proteomes attributed to an order to the total number of PrD-bearing species identified in this order. The highest average numbers of PrDs per species were identified in *Megavirales* and *Herpesvirales* species (Table 2; Supplementary Table 3).

**Table 2:** Mean PrD numbers per species in the same viral order.

Order	Number of PrD-carrying viral species	Total number of PrDs identified in the order	Mean number of PrDs per species
*Herpesvirales*	74	500	6.75
*Megavirales*	78	694	8.86
*Mononegavirales*	35	85	2.42
*Nidovirales*	60	114	1.90
*Picornavirales*	44	60	1.36
*Tymovirales*	16	23	1.43
Unassigned	427	1204	2.83

Next, we evaluated the LLRs in the viral orders and families. The largest number of viruses with the highest LLR scores, over 50 and 40, were identified in the order *Megavirales* (families *Mimiviridae*, *Phycodnaviridae*, and *Poxviridae*), while only a few were obtained in *Herpesviridae*. (Supplementary Tables 4 and 5). By analyzing top 100 scoring PrDs of the viruses with the greatest prion-forming potential, we evaluated the highest LLR scores predominantly among *Megavirales*, *Herpesviridae*, and in viruses of unassigned orders (Supplementary Table 6). Twenty seven percent of these top 100 PrDs were identified in the *Mimiviridae* species, order *Megavirales*, of *Acanthamoeba*, with the mean LLR score of 48.68. We also applied the PAPA prion prediction algorithm to these top 100 scoring PrDs and the majority of the results were consistent with the PLAAC analysis^35,36^.

Additionally, we analyzed the PrD enrichment in the proteomes of different viral species. The highest enrichment rate was found for the members of the *Megavirales* order, with at least five PrDs per proteome in the viruses belonging to the *Mimiviridae* and *Phycodnaviridae* families (Supplementary Table 7). The highest number of different viral species with over 10 PrDs per proteome was found in the *Herpesviridae* family.

### Association of viral PrDs with the functional domains

**Figure 3:**
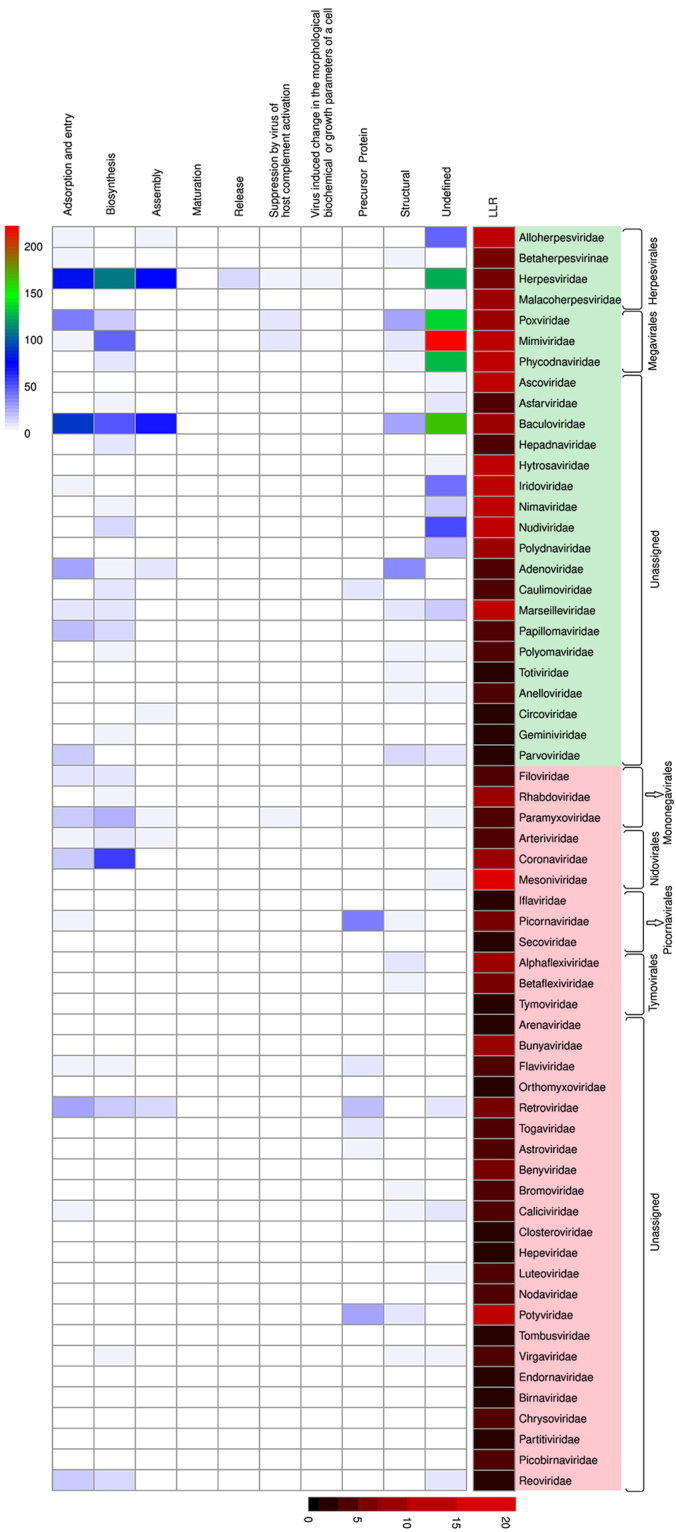
PrD distribution in viral proteins and the GO term analyses. Heatmap of PrD distribution in viral proteins. The correlations between the functions of PrD-containing proteins, PrD numbers, their LLR scores, and viral families are presented. Columns, the main protein function; rows, viral families. Cells indexed by rows and columns are marked using a color gradient, ranging from white (no PrD-containing proteins) to red (maximum number of proteins with PrDs). Mean LLC scores of proteins presented in the far-right column are denoted by using a gradient of red, ranging from black (score 0) to saturated red (score 70; color bar). Viral families are grouped according to their genetic material. Green background, DNA-viruses; red background, RNA-viruses.

We clustered PrDs into six functional groups based on the major processes during the viral interaction with the host cell: adsorption and entry, biosynthesis, including the transcription, translation, and synthesis of viral components, maturation, assembly, release, and a group comprising proteins with an unknown function^49^. We separately analyzed the PrDs in the viral precursor proteins^50^. Additionally, we analyzed the PrDs identified in proteins with the functions not related to the main viral processes, but that, nevertheless, play important roles in disease pathogenesis, the virus-induced changes in the morphological, biochemical, or growth parameters of cells, and the suppression of host complement activation. The correlations the PrDs and protein functions were identified, and the PrD numbers, their LLR scores, and viral families were analyzed (Fig. 3).

To facilitate the interpretation of the results, we grouped the proteins based on their functions using the GO terms (Supplementary Figs 1–10).

Following this, we identified 433 PrDs (medium LLR score, 5.05) in proteins involved in the viral adsorption and entry, and predominantly associated with the host cell-membrane binding. This group contains proteins belonging to different GO terms, including the integral component of membrane, viral envelope, virion attachment to host cell, fusion of virus membrane with host plasma membrane, receptor-mediated virion attachment to host cell, and others (Supplementary Fig. 1). We identified PrDs in proteins associated with the adsorption and those involved in the direct contact with the host cell, such as spike proteins, VP1, glycoproteins, hemagglutinin-neuraminidase, etc.^51^. Heatmap analysis results showed that the members of *Baculoviridae* and *Herpesviridae* have the highest number of PrDs associated with the viral adsorption and entry (Fig. 3). Furthermore, we identified PrDs in glycoproteins and membrane proteins of viruses that affect human health, such as human α-, β-, and γ-herpesviruses (human herpes virus 1, 2, 5, and 7) and other viruses associated with human diseases, such as hepatitis B and C, Marburg virus, rotavirus A, human immunodeficiency virus 1 (HIV 1), and others^52^.

The biggest cluster of PrDs (502 proteins) contained the proteins involved in viral transcription, translation, and protein synthesis (LLR score, 6.69), with multiple molecular functions and belonging to different GO terms. The members of *Herpesviridae* family contained the majority of these PrDs (Fig. 3; Supplementary Table 1; Supplementary Fig. 2). We identified PrDs in the DNA polymerases of different human herpesviruses, such as cytomegalovirus, Epstein-Barr, varicella-zoster viruses, and herpes simplex virus 2. Additionally, we detected them in the Epstein-Barr nuclear antigens (EBNA) and large tegument protein deneddylase of these viruses, in the RNA-directed 5′-3′ RNA polymerases and nucleoproteins of *Filoviridae* viruses, such as Marburg virus and Zaire ebolavirus, in the nucleoproteins of human coronavirus and porcine epidemic diarrhea (PED) virus (*Coronaviridae*), and others^53,54^.

Following this, we analyzed PrD-containing viruses associated with the viral assembly. We identified 209 PrDs with the mean LLR score of 7.79. The main GO terms represented were the viral capsid assembly, serine-type endopeptidase activity, nuclear capsid assembly, viral DNA genome packaging, and others (Supplementary Fig. 3). The key PrD-containing proteins shown to be involved in the viral assembly were identified in the *Baculoviridae* and *Herpesviridae* families. We identified desmoplakin as the main PrD-containing protein in *Baculoviridae*, capsid scaffold protein and small capsomere-interacting protein 1 were the most abundant in different herpesviruses, Gag protein in many *Retroviridae* and other viruses (Fig. 3)^55,56^.

The identified PrDs in proteins involved in the release of viral progeny from the host cell were shown to be less abundant, with only 19 proteins found to contain these domains (LLR score, 3.68). In the GO terms, this group predominantly comprised proteins associated with the DNA packaging and viral release from the host cell. The highest number of them were identified in *Herpesviridae*, including partial proteins, capsid vertex component 2, and tegument protein pp150 (Fig. 3; Supplementary Fig. 4).

Additionally, we identified six PrDs in proteins associated with the viral maturation (LLR score, 23.61) and with the GO terms associated with the integral components of the membrane and methyltransferase activity in different viruses (Fig. 3; Supplementary Fig. 5)^57^.

The PrDs were also detected in 223 structural proteins, predominantly represented by capsid, coat proteins, and hexons (mean LLR score, 5.78)^58^. Notably, the majority of these proteins were found in different non-enveloped viruses, primarily from the *Adenoviridae* and *Baculoviridae* families, and these were less abundant in the enveloped viruses, primarily belonging to *Poxviridae* (Supplementary Fig. 6).

Furthermore, we identified 138 PrDs (mean LLR score, 6.47) in the viral precursor proteins. Positive-strand RNA viruses are characterized by a positive strand RNA genome encoding a single poly-protein precursor, which, during the post-translational processing, are cleaved and processed into the mature proteins. We identified PrDs in the genome polyproteins of *Picornavirales* (foot-and-mouth disease virus, enterovirus B, and cardiovirus B) and *Flaviviridae (*Zika virus, hepatitis C virus), in the Gag polyprotein of *Retroviridae* (HIV1, bovine leukemia virus), and others (Fig. 3, Supplementary Table 7)^59,60^.

The PrDs identified in the proteins associated with the viral suppression of host complement activation were less abundant, and only 39 of these proteins were identified (mean LLR score, 7.11). In the GO terms, they were represented with the G-protein coupled receptor activity, evasion or tolerance of host immune response, metal ion binding, and unassigned processes. PrDs were found in NF-kappa B inhibitors, envelope glycoprotein UL33, ankyrin repeat-containing protein, and others, and among different viruses, including some important human pathogens, such as cytomegalovirus, Kaposi’s sarcoma-associated herpesvirus, and HIV1 and 2 (Supplementary Fig. 8)^61,62^.

We found 14 PrDs (mean LLR score, 11.09) in proteins implicated in the virus-induced change in the morphological, biochemical, or growth parameters of cells. Among these, we identified late membrane protein 1 and K1 in Epstein-Barr virus and Kaposi’s sarcoma-associated herpesvirus (Fig. 3; Supplementary Fig. 9)^63^.

Finally, we identified 1097 PrDs in proteins with still unknown functions (mean LLR score, 9.79). The vast majority of these are uncharacterized proteins, which has still not been reviewed in the GO terms (Supplementary Fig. 10).

## Discussion

This study is the most complete evaluation of PrDs among viruses except for the bacteriophages^34^. The results of our study highlight some previously overlooked viral characteristics that may play important roles in viral infections. We determined that more PrDs can be found in the DNA-viruses compared with their numbers in the RNA-viruses, and in the enveloped viruses, compared with that in the non-enveloped ones. This may be partially explained by the larger genome size and protein numbers in DNA-viruses^64^.

We identified PrDs in functionally distinct proteins of different viral orders, indicating that these PrDs are conserved in different viruses. However, the PrDs were not identified in all viral families and species. Our analyses demonstrated that only approximately 23% of all analyzed viral proteomes available in public databases contain at least one PrD, suggesting that the presence of PrD-containing proteins may be beneficial, but not obligatory. We identified PrDs in many human viral pathogens, but other viruses affecting human health were shown to have a few or no PrDs in their proteomes, such as hepatitis A, E, and D viruses, papillomaviruses, some members of *Orthomyxoviridae*, and others.

At the order level, we showed that the PrDs are more frequent among *Megavirales* and *Herpesvirales*, while, at the species level, the highest number of PrDs was found in *Acanthamoeba polyphaga* mimivirus, *Paramecium bursaria* Chlorella virus NY2A, *Acanthamoeba castellanii* mamavirus *(Megavirales*), and *Heliothis zea nudivirus* (unassigned order). Among human pathogens, the highest prevalence of PrD was found in cytomegalovirus and Epstein-Barr virus (*Herpesvirales*) and HIV1 (*Retroviridae* family, unassigned order) (Supplementary Table 7).

The analysis of the top 100 scoring PrDs, with the highest number of QN-rich domains, they were found to be most common among *Mimiviridae*, which infect *Acanthamoeba*, and *Phycodnaviridae*, which infect algae and belong to the *Megavirales*. Of these, only some proteins were *Herpesvirales* proteins, while the majority of them was shown to be identified in the viruses of the unassigned order. No human viruses were shown to have LLR scores over 31 and none were represented in the top 100 LLR-scoring group. The majority of these proteins has not been characterized, with still unknown functions, and therefore, the functional relevance of these findings remains unclear. We also applied the PAPA prion prediction algorithm to these top 100 scoring PrDs^35,36^. The majority of data received by the PAPA, was overlapping with PLAAC algorithm, which is in agreement with previous studies, showing that PLAAC and PAPA commonly overlap as both programs were trained on prion proteins of *S. cerevisiae*^38^. However, there were certain differences, and some PrDs that had high PLAAC score were found to be below the 0.05 cutoff of PAPA algorithm. Such a discrepancy can be explained by the fact that although both of these programs have a high level of prediction accuracy for prion and non-prion protein determination, the precision of the analysis is below 100%^35,36^.

The order *Megavirales* is a recently established order that comprises of diverse group of the DNA-viruses infecting eukaryotic hosts, which are characterized by large genomes, almost 10 times larger than those of the *Herpesviridae*^48^. Here, DNA-viruses were found to harbor more high-scoring prions, as expected, but the high LLR scores obtained for these viruses is not due to the longer amino-acid sequences, but to the increased presence of QN-residues.

Furthermore, we aimed to determine the correlation between the PrD-containing protein functions and the frequency of PrDs in the viral proteomes found in different viral families. Adhesion and entry of viral nucleic acids represent crucial steps in the viral-host interactions and the viral PrD-containing proteins showed to be involved in these processes represented the second largest group. We identified the PrDs in the viral surface proteins that are involved in the direct contact and fusion of viruses with the host cell membrane, indicating that PrDs may be functionally implicated in these processes as well. Moreover, a similar trend was previously noted in the distribution of PrDs in proteins responsible for the bacterial and bacteriophage interactions^39^. Interestingly, most PrDs were found in *Baculoviridae*, the rod-shaped viruses. We identified PrDs in 56 out of 66 known species belonging this family, indicating that this high prevalence of PrDs in one viral family may not be a coincidence. Therefore, we further showed that one of the PrDs associated with the cell adhesion and entry most frequently found in *Baculoviridae* is occlusion-derived virus envelope protein 66 (ODV-E66), which was recently the first identified viral chondroitin lyase, an enzyme that degrades chondroitin and is associated with viral entry^65,66^.

Of 543 PrDs found to be associated with the viral interaction with the host cells, only four proteins were identified in the plant viruses (potato mop-top virus, Dasheen mosaic virus, only Syngen Nebraska virus 5, and Fiji disease virus), while none were identified in the fungi viruses. This may be explained by the presence of cell wall in the plant and fungal cells, requiring different mechanism of viral entry^67^. Plant viruses are known to have no specific mechanisms of entry, but instead they take advantage of the plant injury, vectors such as insects, or through a cell-to-cell movement of viral progeny in the infected plant^68,69^. Taken together, the presented findings, showing that PrDs are present in the proteins of animal viruses that interact with cell membranes, and their absence from the plant and fungal viruses indicates that the identified PrDs associated with adhesion and entry may have important functional roles.

Proteins involved in the viral biosynthesis were shown to harbor PrDs as well. We found numerous PrDs in different nucleic acid-binding proteins, proteins with kinase activity, regulators of chromosome condensation, and others. Several PrDs were found in the DNA and RNA polymerases, helicases, and EBNA1. However, the roles of PrDs in the viral proteins associated with biosynthesis remain unclear, but they may be necessary for the efficient protein-nucleic acid interactions. Similar trend was observed in the eukaryotes, where the most abundant PrDs can be found in the nucleic acid-binding proteins^70^. In humans, the QN-biased amino acid-enriched regions are frequently found in the RNA-binding proteins and regulatory molecules^66^. These nucleic acid-binding prion candidates are found in the proteins associated with neurodegenerative disorders, such as amyotrophic lateral sclerosis (ALS), Alzheimer’s disease, and Huntington’s disease, and they have been suggested to act as the epicenters of misfolding^71^.

Some viruses may be implicated in the prion misfolding in humans since it was observed that the *de novo* appearance of prions can be facilitated by another PrD-containing protein^72^. Previously, human herpes virus was shown to be associated with the development of Alzheimer’s disease, and several epidemiological studies demonstrated the presence of HSV1 antigens in the cerebrospinal fluid of Alzheimer’s disease patients^73,74^. It can be speculated that the presence of multiple PrDs identified in this study in the HSV1 proteins, including nucleic acid-binding and surface proteins, may represent a seeding trigger of the protein misfolding in Alzheimer’s disease. However, further experiments are required to confirm this.

Moreover, we detected some interesting trends in the PrD distribution among other functional groups of viral proteins. The presence of PrDs in viral assembly proteins is most likely important for the capsid assembly, which is known to be characterized by the nucleation-and-growth mechanism, dependent on the capsid assembly kinetics^75^. Therefore, the glutamine-rich motifs, which mediate protein-protein interactions and have greater potential for the kinetic conformational diversity, in capsid assembly proteins may determine the pathways of capsid protein complex assembly^76^. The majority of PrDs in the assembly proteins were found in the enveloped viruses, while PrDs in the structural proteins were predominantly identified in the non-enveloped ones. Further analyses indicated the PrD enrichment in the assembly proteins, such as adenoviral major capsid proteins hexons, found in different members of *Adenoviridae*, including human adenovirus serotype 40 (AdV40)^77^.

Adenoviral hexons are known to play multiple roles including structural roles and those associated with the host-cell interactions. The identified PrDs, according to PLAAC analysis, are located within 130–167 amino acids in the AdV40, which partially corresponds to a variable region V1 located in the AdV40 serotype-specific loop 1^78,79^. These hypervariable regions are located on the surface of hexons, and the loops protruding from the capsid are thought to be implicated in the immune response triggering through the interactions with the human adenovirus-specific T-cells^68,80,81^.

Another thing that caught our attention in terms of viral assembly and prionogenic properties is that in eukaryotes, PrDs are known to be implicated in liquid-liquid phase separation (LLPS)^82–85^. LLPS is known to be an important process in the nucleation and growth of protein crystals and is suggested as the first step for viral capsid growth^86,87^. Therefore, the discovery of PrDs in proteins associated with viral capsid growth indicates a possible role of these prionogenic properties in viral assembly. An indirect reference to that is that human cytomegalovirus capsid scaffolding protein, which plays an essential structural role in assembling the viral capsid, was identified in the current study as having a PrD, and in recent work by Vernon, R. *et al*., it was shown to be associated with phase separation^88^.

In viruses, the presence of PrDs can be associated with the precursor proteins of positive-sense RNA viruses, such as *Picornavirales* and *Retroviridae*. By analyzing this group, we detected the PrDs in some precursor proteins, which were not found in the functional proteins cleaved from these primary polyproteins of *Picornavirales*. Only enterovirus B were shown to have the PrDs in both polyprotein and capsid protein VP1. These observations require additional investigations, since they cannot be due to an incomplete analysis, as the UniProt database contains the data on many *Picornavirales* proteins. It is likely that certain post-translational modifications of precursor polyproteins may result in the removal of the PrDs during the process of maturation.

Furthermore, PrD-containing proteins were found to be associated with the suppression of host complement activation and virus-induced changes in cells, including the modulation of host apoptotic process^82^. We identified the PrDs in these proteins in many unrelated viruses of different hosts. The PrDs were primarily identified in human and insect viruses with demonstrated ability to establish persistent infections: *Baculoviridae*, HIV1, and *Herpesviridae* species, such as HSV1, Epstein-Barr virus, cytomegalovirus, and oncogenic HSV8^89,90^. This indicates that the PrDs in these proteins may be implicated in the establishment of persistent viral infections and affect the adaptive immune response.

Taken together, we identified numerous putative PrD-containing proteins in viruses. Although we used a low threshold of PLAAC score for identification (or in other words even proteins with a low probability of being a prion were included in the analysis), there were still over 650 proteins with high LLR score over 10, which were the most promising prion candidates (Supplementary Table 8). We observed consistent PrD distribution patterns in different viral families and species, and these domains were identified in a variety of proteins. However, since the majority of viruses were shown to lack the PrDs, this shows that the presence of PrDs is beneficial, but not obligatory, which agrees with the results obtained for the PrDs found in bacteria and bacteriophages^32,34^. Further analyses are required to elucidate the role of the identified PrDs in viral proteins, primarily those found in the human viral pathogens.

The predictive approach employed in this study revealed for the first time a large set of putative PrDs in numerous proteins of the emerging human viral pathogens, including those associated with persistent viral infections, oncogenic processes, hemorrhagic fevers, and others. Further analyses of these PrD-containing proteins may improve our understanding of viral infections, and they need to be further expanded along with the discovery of novel viral species under the Virome project^91–94^.

## Electronic supplementary material

LLR score showing the predicted putative PrDs across viruses

Summary of the LLR score of prion predictions across viral orders

Mean PrD numbers per species in the same viral order

Distribution of viral orders families with the LLR scores higher than 40, 50, and 60

Distribution of viral families with the LLR scores higher than 40, 50, and 60

Top 100 scoring PrDs in different viral species

PrD enrichment rate in different viral species

Distribution of different viral species with LLR scores higher than 10

Clustering of viral PrD-containing proteins according to their GO terms
