# Supplementary material for: Prion-like Domains in Eukaryotic Viruses
Source: Sci Rep. 2018 Jun 12;8:8931. doi: 10.1038/s41598-018-27256-w (PMC5997743; doi:10.1038/s41598-018-27256-w)
Supplement: Supplementary file 2 — Summary of the LLR score of prion predictions across viral orders [file 41598_2018_27256_MOESM2_ESM.pdf]

Prion-like Domains in Eukaryotic Viruses

George Tetz, Victor Tetz

Supplementary Table 2. Summary of the LLR score of prion predictions across viral orders.

							Dunn test				
**virus_order**	**N Obs**	**Mean**	**Std Dev**	**Minimum**	**Maximum**	**Median**	**Herpesvirales**	**Megavirales**	**Mononegavirales**	**Picornavirales**	**Tymovirales**
Herpesvirales	500	6.74	7.73	0.02	63.80	4.24					
Megavirales	694	10.35	11.27	0.02	74.68	6.42	<.0001				
Mononegavirales	75	3.93	5.66	0.16	41.38	2.39	0.0109	<0.0001			
Nidovirales	114	7.92	9.14	0.07	42.95	5.06	0.8978	0.1157	0.0059		
Picornavirales	60	5.98	5.71	0.10	26.98	3.53	1	0.0406	0.2115	0.9579	
Tymovirales	23	6.43	5.17	0.07	16.83	5.91	0.9944	0.8398	0.2216	1	0.9926
Unassigned	1204	6.91	8.03	0.00	51.14	4.11					

Nonparametric analysis of variances $F_{5,296}=27.63$; $p<.0001$

Pairwise comparison Dunn test
