# Supplementary material for: Prion-like Domains in Eukaryotic Viruses
Source: Sci Rep. 2018 Jun 12;8:8931. doi: 10.1038/s41598-018-27256-w (PMC5997743; doi:10.1038/s41598-018-27256-w)
Supplement: Supplementary file 3 — Mean PrD numbers per species in the same viral order [file 41598_2018_27256_MOESM3_ESM.pdf]

Prion-like Domains in Eukaryotic Viruses

George Tetz, Victor Tetz

Supplementary Table 3. Mean PrD numbers per species in the same viral order.

								Dunn test				
virus_order	N Obs	Sum	Mean	Std Dev	Minimum	Maximum	Median	Herpesvirales	Megavirales	Mononegavirales	Nidovirales	Picornavirales
Herpesvirales	74	500	6,85	6,68	1	30	4					
Megavirales	78	694	9,51	18,81	1	124	4	0,8753				
Mononegavirales	35	85	2,21	2,12	1	11	1	<.0001	0,0003			
Nidovirales	60	114	1,87	2	1	11	1	<.0001	<.0001	0,8166		
Picornavirales	44	60	1,36	1,2	1	7	1	<.0001	<.0001	0,0965	0,5452	
Tymovirales	16	23	1,44	0,63	1	3	1	<.0001	0,0002	0,9148	1	0,914
Unassigned	427	1204	2,78	3,44	1	38	1					

Nonparametric analysis of variances $F_{5,296}$=27.63; p<.0001

Pairwise comparison Dunn test
