# Supplementary material for: Prion-like Domains in Eukaryotic Viruses
Source: Sci Rep. 2018 Jun 12;8:8931. doi: 10.1038/s41598-018-27256-w (PMC5997743; doi:10.1038/s41598-018-27256-w)
Supplement: Supplementary file 4 — Distribution of viral orders families with the LLR scores higher than 40, 50, and 60 [file 41598_2018_27256_MOESM4_ESM.pdf]

Prion-like Domains in Eukaryotic Viruses

George Tetz, Victor Tetz

Supplementary Table 4. Distribution of viral orders families with the LLR scores higher than 40, 50, and 60.

Table of virus_order by LLR_cod							
		LLR_cod					Total
		<30	>30	>40	>50	>60	
**virus_order**							
**Herpesvirales**	**Frequency**	491	6	2	0	1	500
	**Col Pct**	19.07	9.38	7.69	0	50	
**Megavirales**	**Frequency**	638	29	10	9	1	687
	**Col Pct**	24.78	45.31	38.46	69.23	50	
**Mononegavirales**	**Frequency**	84	0	1	0	0	85
	**Col Pct**	3.26	0	3.85	0	0	
**Nidovirales**	**Frequency**	107	5	1	0	0	113
	**Col Pct**	4.16	7.81	3.85	0	0	
**Picornavirales**	**Frequency**	60	0	0	0	0	60
	**Col Pct**	2.33	0	0	0	0	
**Tymovirales**	**Frequency**	23	0	0	0	0	23
	**Col Pct**	0.89	0	0	0	0	
**undef**	**Frequency**	1172	24	12	4	0	1212
	**Col Pct**	45.51	37.5	46.15	30.77	0	
**Total**	**Frequency**	2574	64	26	13	2	2679
