# Supplementary material for: Prion-like Domains in Eukaryotic Viruses
Source: Sci Rep. 2018 Jun 12;8:8931. doi: 10.1038/s41598-018-27256-w (PMC5997743; doi:10.1038/s41598-018-27256-w)
Supplement: Supplementary file 5 — Distribution of viral families with the LLR scores higher than 40, 50, and 60 [file 41598_2018_27256_MOESM5_ESM.pdf]

Prion-like Domains in Eukaryotic Viruses

George Tetz, Victor Tetz

Supplementary Table 5. Distribution of viral families with the LLR scores higher than 40, 50, and 60.

Table of virus_family by LLR_cod							
		LLR_cod					Total
		<30	>30	>40	>50	>60	
virus_family							
Adenoviridae	Frequency	80	0	0	0	0	80
	Col Pct	3.11	0	0	0	0	
Alloherpesviridae	Frequency	54	2	1	0	0	57
	Col Pct	2.1	3.13	3.85	0	0	
Alphaflexiviridae	Frequency	15	0	0	0	0	15
	Col Pct	0.58	0	0	0	0	
Anelloviridae	Frequency	11	0	0	0	0	11
	Col Pct	0.43	0	0	0	0	
Arenaviridae	Frequency	1	0	0	0	0	1
	Col Pct	0.04	0	0	0	0	
Arteriviridae	Frequency	24	0	0	0	0	24
	Col Pct	0.93	0	0	0	0	
Ascoviridae	Frequency	5	1	0	0	0	6
	Col Pct	0.19	1.56	0	0	0	
Asfarviridae	Frequency	17	0	0	0	0	17
	Col Pct	0.66	0	0	0	0	
Astroviridae	Frequency	13	0	0	0	0	13
	Col Pct	0.5	0	0	0	0	
Baculoviridae	Frequency	385	11	3	0	0	399
	Col Pct	14.95	17.19	11.54	0	0	
Baculoviridae	Frequency	4	0	0	0	0	4
	Col Pct	0.16	0	0	0	0	
Benyviridae	Frequency	7	0	0	0	0	7
	Col Pct	0.27	0	0	0	0	
Betaflexiviridae	Frequency	7	0	0	0	0	7
	Col Pct	0.27	0	0	0	0	
Betaherpesvirinae	Frequency	18	0	0	0	0	18
	Col Pct	0.7	0	0	0	0	
Birnaviridae	Frequency	2	0	0	0	0	2
	Col Pct	0.08	0	0	0	0	
Bromoviridae	Frequency	8	0	0	0	0	8
	Col Pct	0.31	0	0	0	0	
Bunyaviridae	Frequency	3	0	0	0	0	3
	Col Pct	0.12	0	0	0	0	
Caliciviridae	Frequency	26	0	0	0	0	26
	Col Pct	1.01	0	0	0	0	
Caulimoviridae	Frequency	20	0	0	0	0	20
	Col Pct	0.78	0	0	0	0	
Chrysoviridae	Frequency	1	0	0	0	0	1
	Col Pct	0.04	0	0	0	0	
Circoviridae	Frequency	9	0	0	0	0	9
	Col Pct	0.35	0	0	0	0	
Closteroviridae	Frequency	6	0	0	0	0	6
	Col Pct	0.23	0	0	0	0	

Coronaviridae	Frequency	74	4	1	0	0	79
	Col Pct	2.87	6.25	3.85	0	0	
Endornaviridae	Frequency	3	0	0	0	0	3
	Col Pct	0.12	0	0	0	0	
Filoviridae	Frequency	23	0	0	0	0	23
	Col Pct	0.89	0	0	0	0	
Flaviviridae	Frequency	21	0	0	0	0	21
	Col Pct	0.82	0	0	0	0	
Geminiviridae	Frequency	8	0	0	0	0	8
	Col Pct	0.31	0	0	0	0	
Hepadnaviridae	Frequency	17	0	0	0	0	17
	Col Pct	0.66	0	0	0	0	
Hepeviridae	Frequency	2	0	0	0	0	2
	Col Pct	0.08	0	0	0	0	
Herpesviridae	Frequency	412	4	1	0	1	418
	Col Pct	16	6.25	3.85	0	50	
Hytrosaviridae	Frequency	10	1	0	0	0	11
	Col Pct	0.39	1.56	0	0	0	
Iflaviridae	Frequency	3	0	0	0	0	3
	Col Pct	0.12	0	0	0	0	
Iridoviridae	Frequency	45	2	3	1	0	51
	Col Pct	1.75	3.13	11.54	7.69	0	
Luteoviridae	Frequency	8	0	0	0	0	8
	Col Pct	0.31	0	0	0	0	
Malacoherpesviridae	Frequency	7	0	0	0	0	7
	Col Pct	0.27	0	0	0	0	
Marseilleviridae	Frequency	46	0	0	0	0	46
	Col Pct	1.79	0	0	0	0	
Mesoniviridae	Frequency	9	0	0	0	0	9
	Col Pct	0.35	0	0	0	0	
Mimiviridae	Frequency	270	15	8	7	0	300
	Col Pct	10.49	23.44	30.77	53.85	0	
Nimaviridae	Frequency	22	0	1	1	0	24
	Col Pct	0.85	0	3.85	7.69	0	
Nodaviridae	Frequency	4	0	0	0	0	4
	Col Pct	0.16	0	0	0	0	
Nudiviridae	Frequency	59	8	4	0	0	71
	Col Pct	2.29	12.5	15.38	0	0	
Orthomyxoviridae	Frequency	3	0	0	0	0	3
	Col Pct	0.12	0	0	0	0	
Papillomaviridae	Frequency	37	0	0	0	0	37
	Col Pct	1.44	0	0	0	0	
Paramyxoviridae	Frequency	55	0	0	0	0	55
	Col Pct	2.14	0	0	0	0	
Partitiviridae	Frequency	2	0	0	0	0	2
	Col Pct	0.08	0	0	0	0	
Parvoviridae	Frequency	43	0	0	0	0	43
	Col Pct	1.67	0	0	0	0	
Phycodnaviridae	Frequency	138	8	1	0	0	147
	Col Pct	5.36	12.5	3.85	0	0	
Picobirnaviridae	Frequency	1	0	0	0	0	1
	Col Pct	0.04	0	0	0	0	
Picornaviridae	Frequency	54	0	0	0	0	54
	Col Pct	2.1	0	0	0	0	
Polydnaviridae	Frequency	24	0	0	0	0	24

	Col Pct	0.93	0	0	0	0	
Polyomaviridae	Frequency	17	0	0	0	0	17
	Col Pct	0.66	0	0	0	0	
Polyomaviridae	Frequency	3	0	0	0	0	3
	Col Pct	0.12	0	0	0	0	
Potyviridae	Frequency	36	2	0	2	0	40
	Col Pct	1.4	3.13	0	15.38	0	
Poxviridae	Frequency	220	5	2	2	1	230
	Col Pct	8.54	7.81	7.69	15.38	50	
Reoviridae	Frequency	44	0	0	0	0	44
	Col Pct	1.71	0	0	0	0	
Retroviridae	Frequency	87	0	0	0	0	87
	Col Pct	3.38	0	0	0	0	
Rhabdoviridae	Frequency	6	0	1	0	0	7
	Col Pct	0.23	0	3.85	0	0	
Secoviridae	Frequency	3	0	0	0	0	3
	Col Pct	0.12	0	0	0	0	
Togaviridae	Frequency	13	0	0	0	0	13
	Col Pct	0.5	0	0	0	0	
Tombusviridae	Frequency	3	0	0	0	0	3
	Col Pct	0.12	0	0	0	0	
Totiviridae	Frequency	6	0	0	0	0	6
	Col Pct	0.23	0	0	0	0	
Tymoviridae	Frequency	1	0	0	0	0	1
	Col Pct	0.04	0	0	0	0	
Virgaviridae	Frequency	19	0	0	0	0	19
	Col Pct	0.74	0	0	0	0	
undef	Frequency	1	1	0	0	0	2
	Col Pct	0.04	1.56	0	0	0	
Total	Frequency	2574	64	26	13	2	2679
