# Supplementary material for: Prion-like Domains in Eukaryotic Viruses
Source: Sci Rep. 2018 Jun 12;8:8931. doi: 10.1038/s41598-018-27256-w (PMC5997743; doi:10.1038/s41598-018-27256-w)

Prion-like domains in eukaryotic viruses

George Tetz, Victor Tetz

Supplementary Fig 1

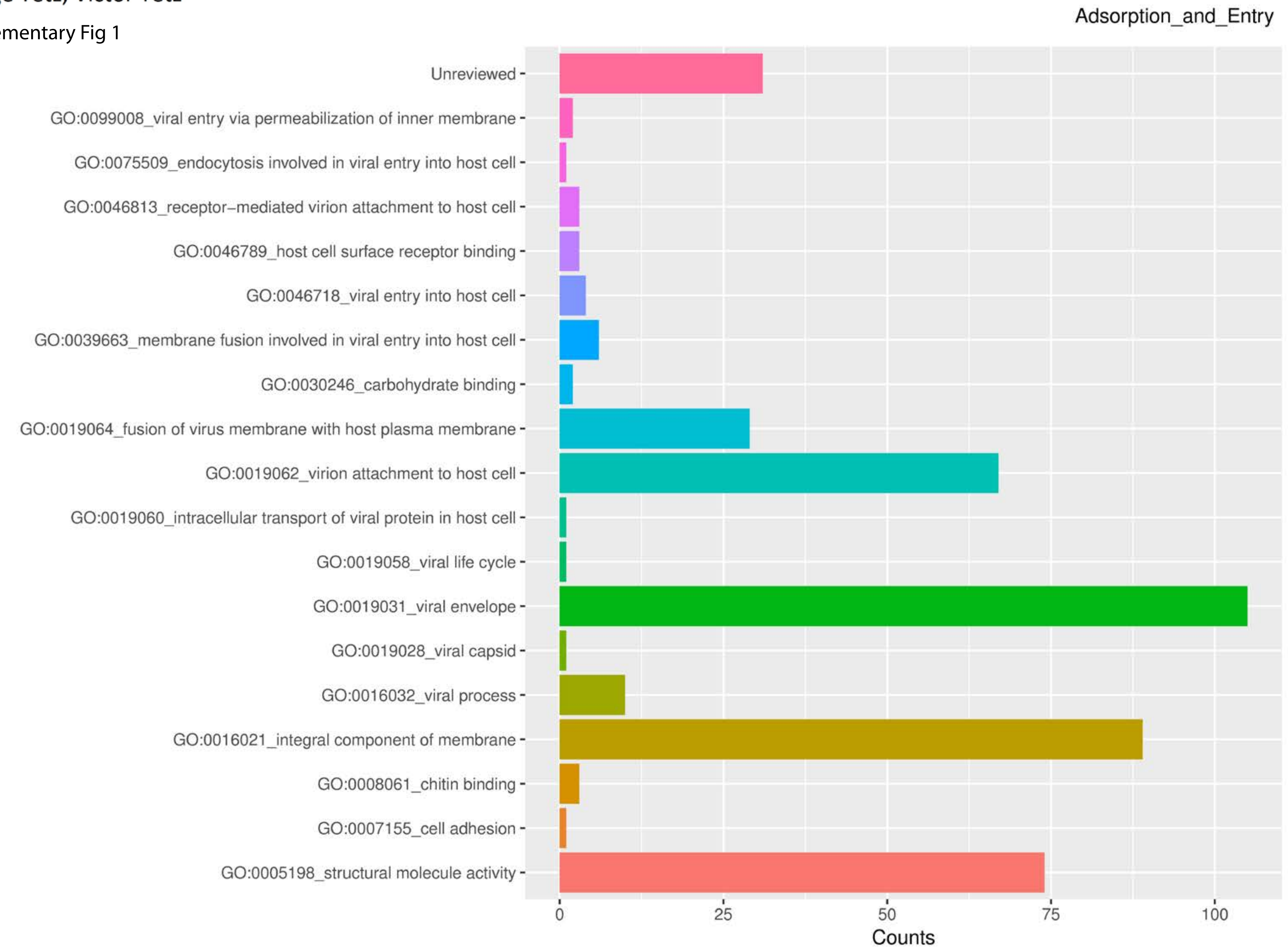

Supplementary Fig 2

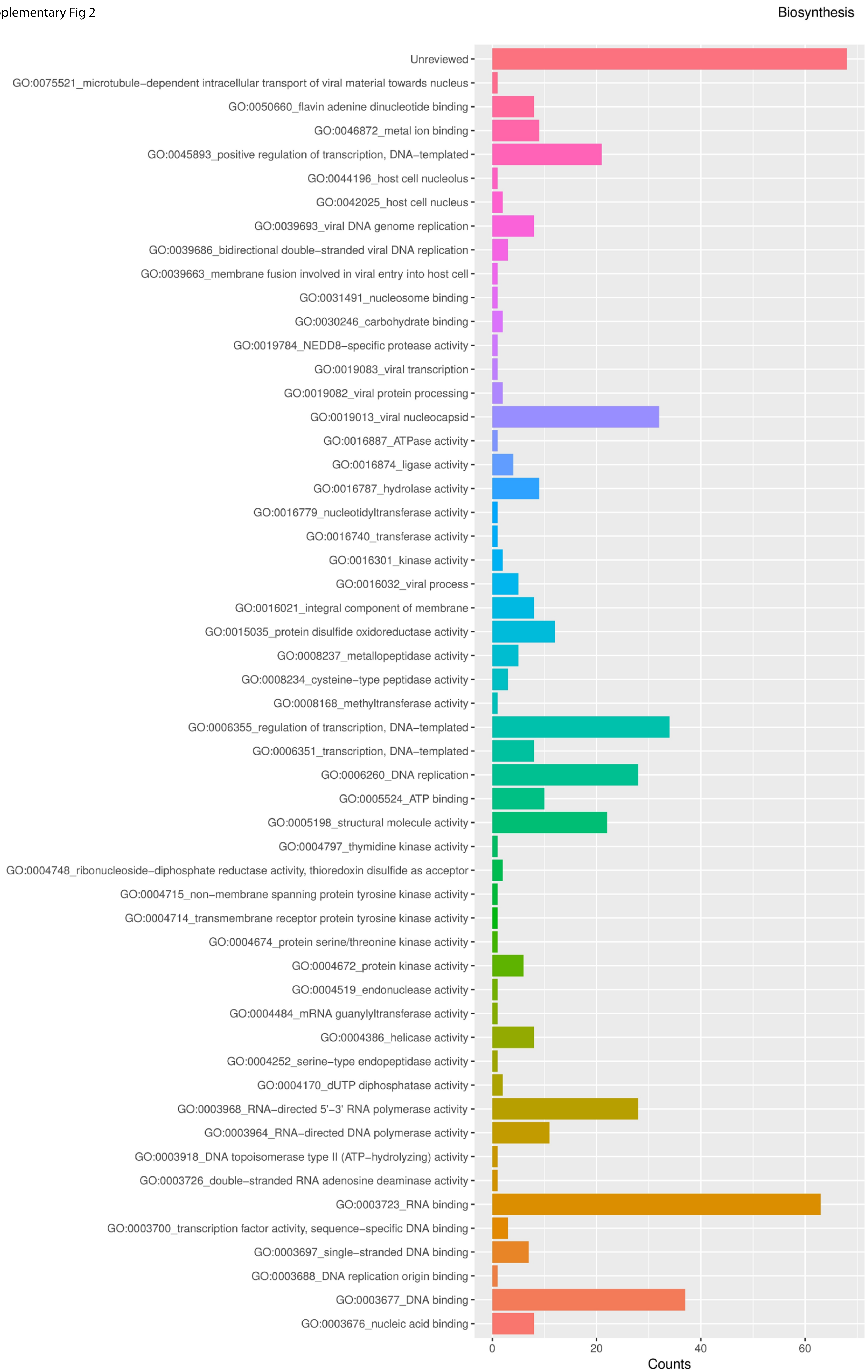

Supplementary Fig 3

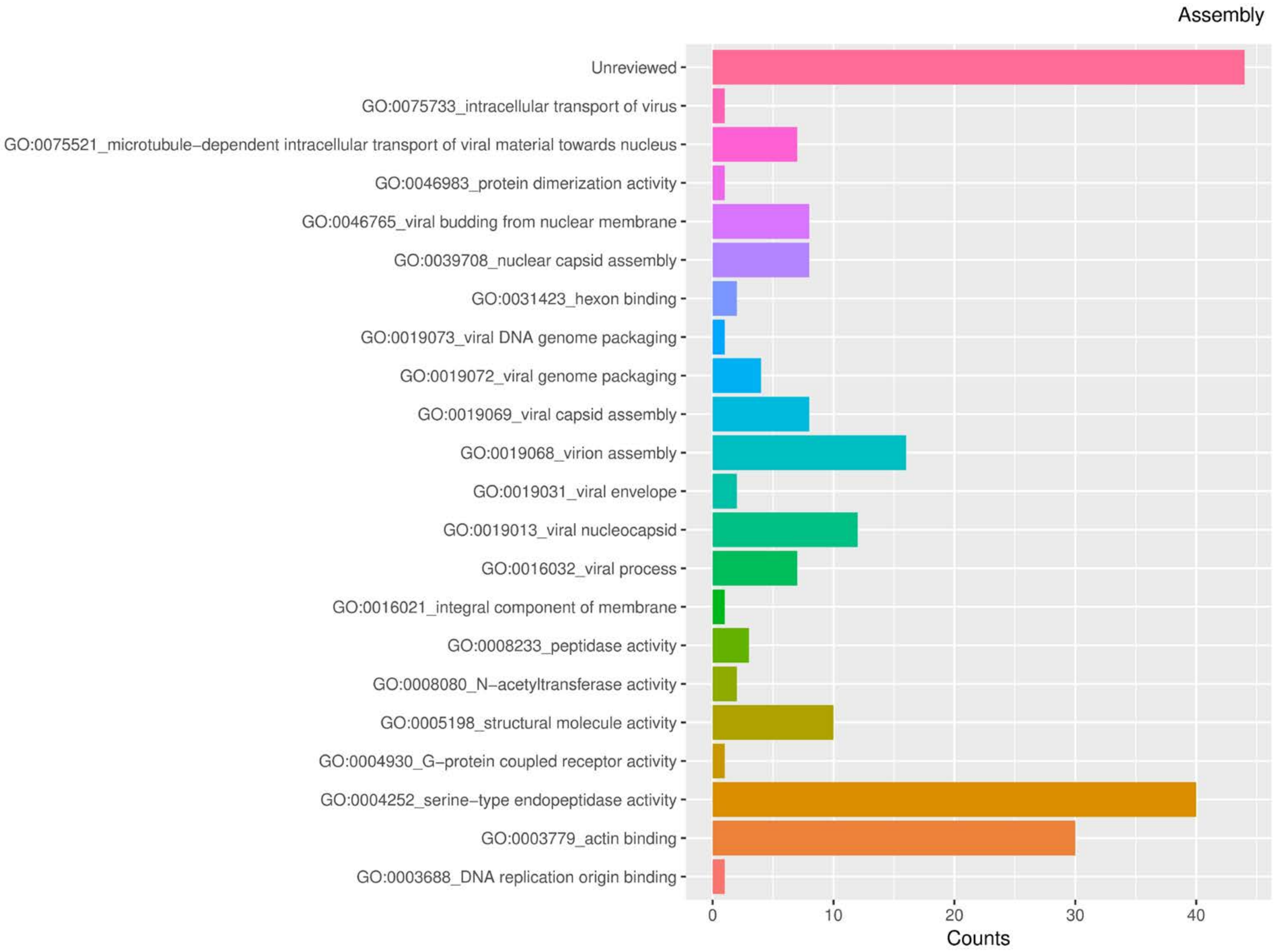

Supplementary Fig 5

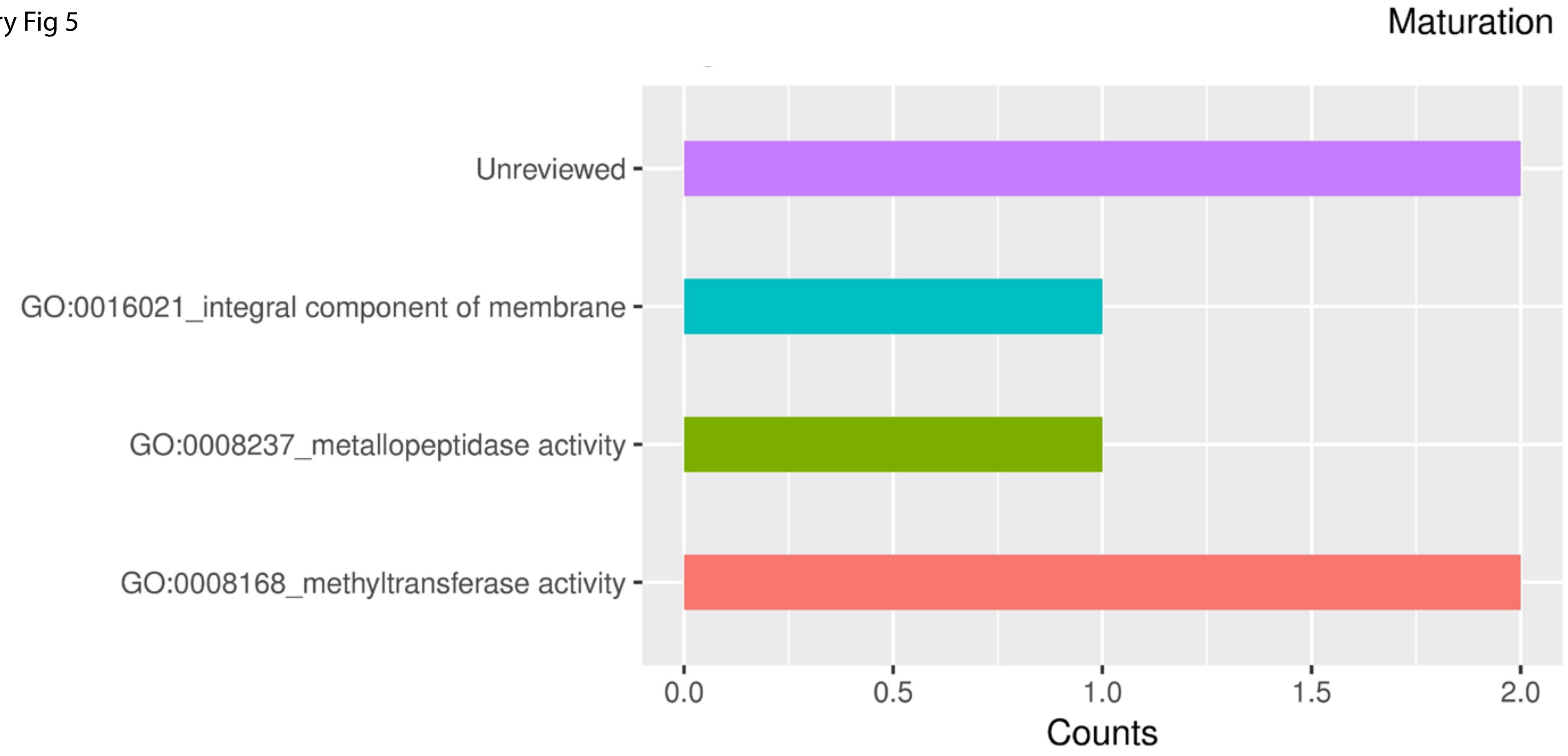

Supplementary Fig 6

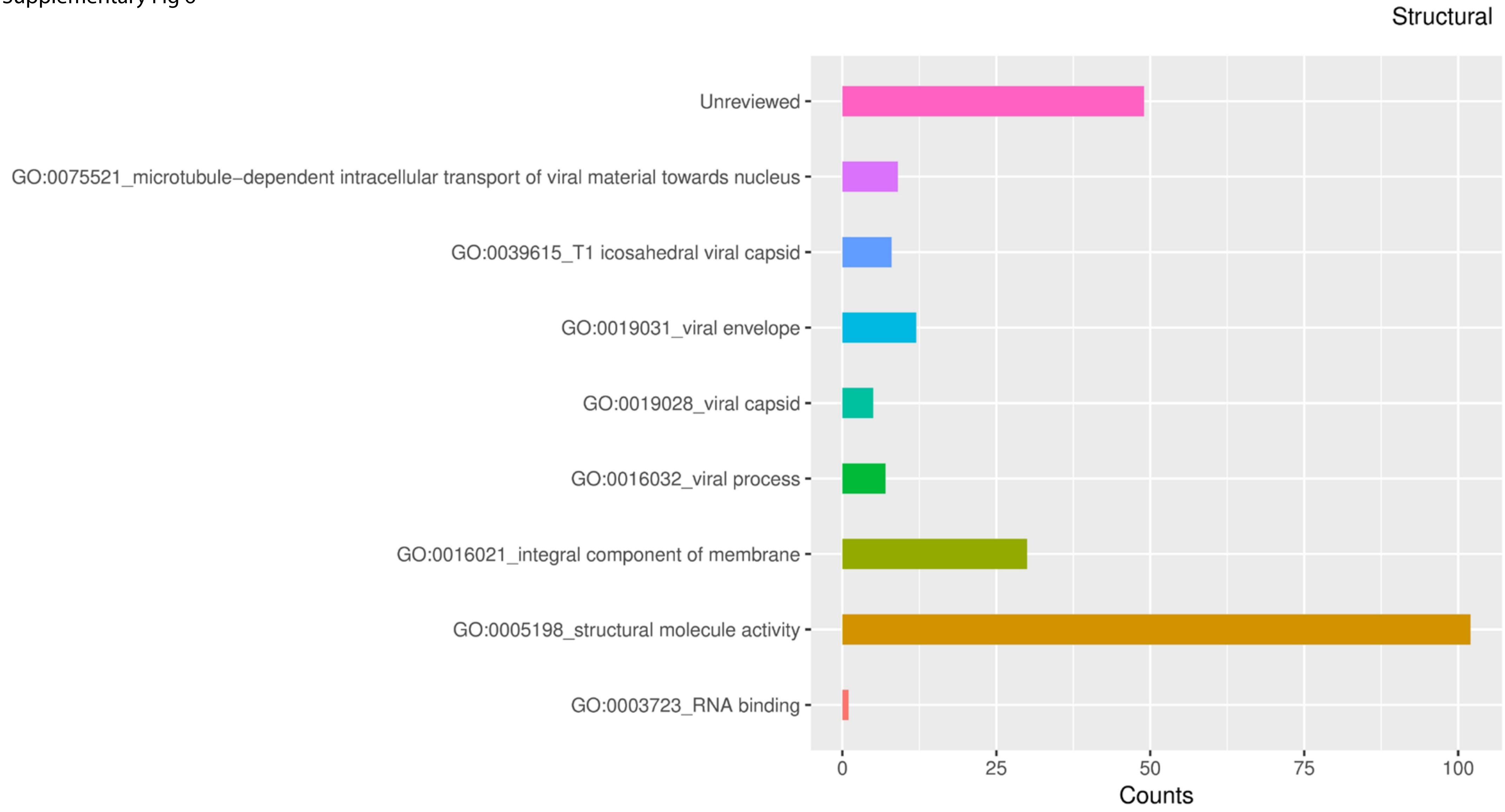

Supplementary Fig 7

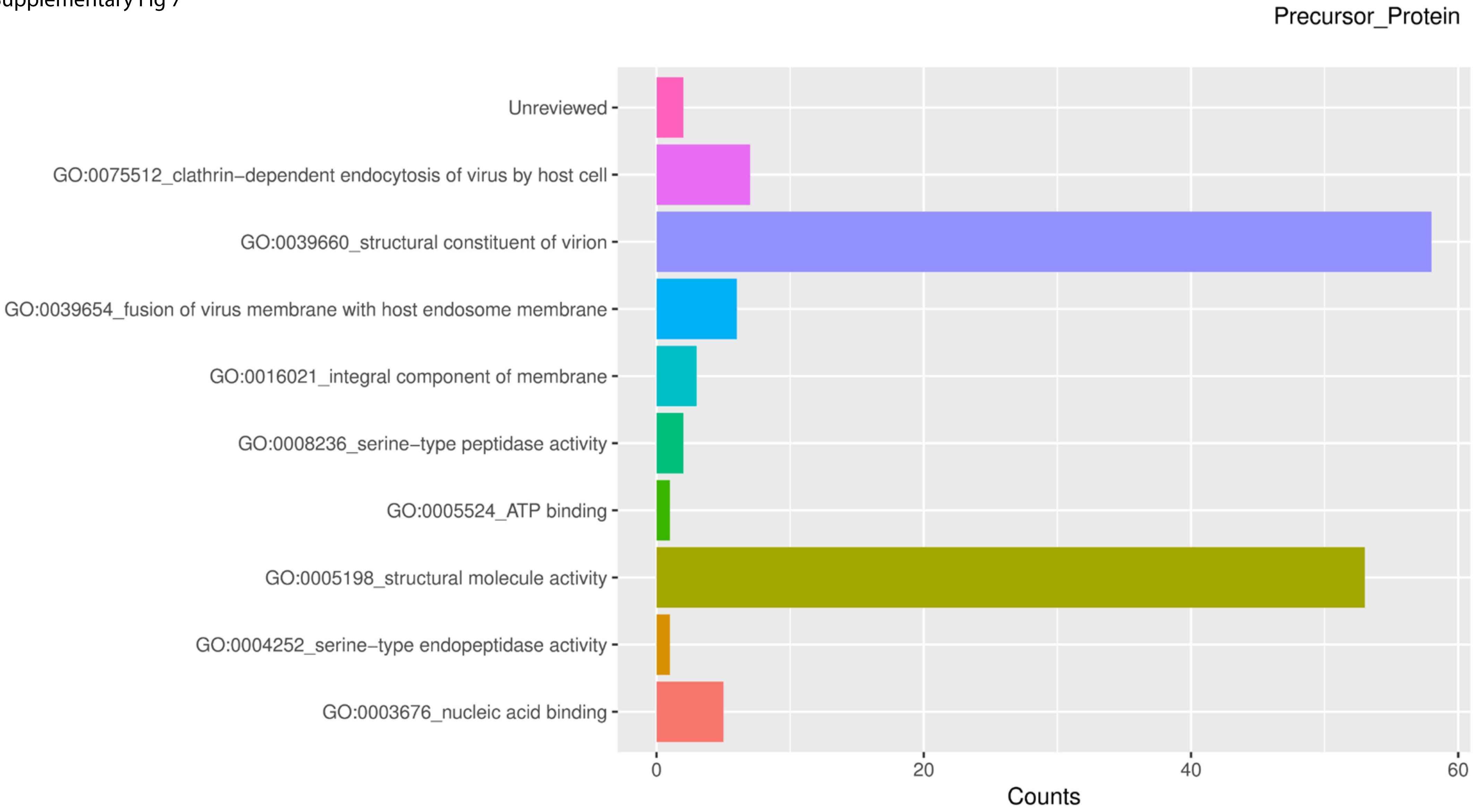

Supplementary Fig 8

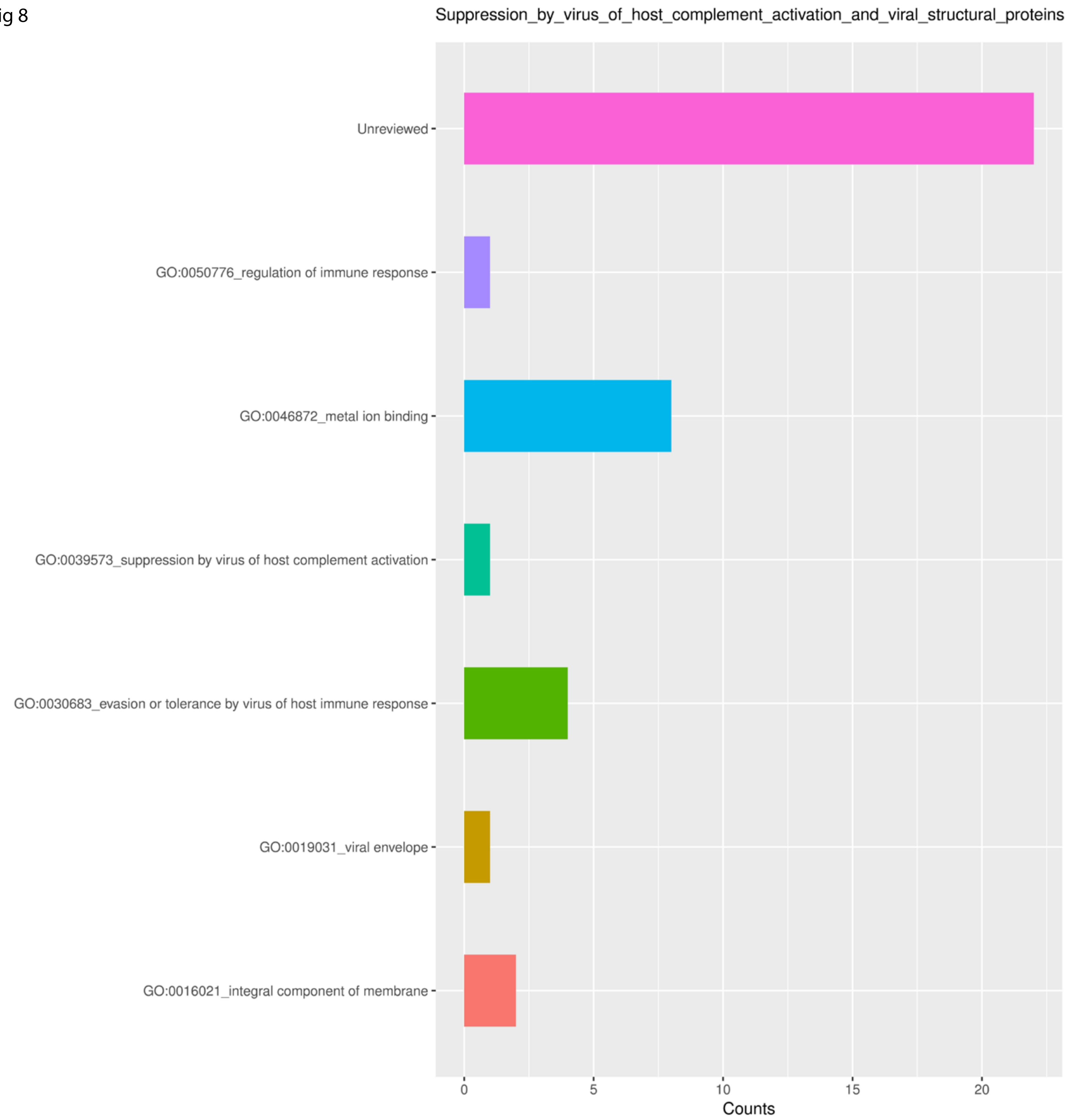

Supplementary Fig 9

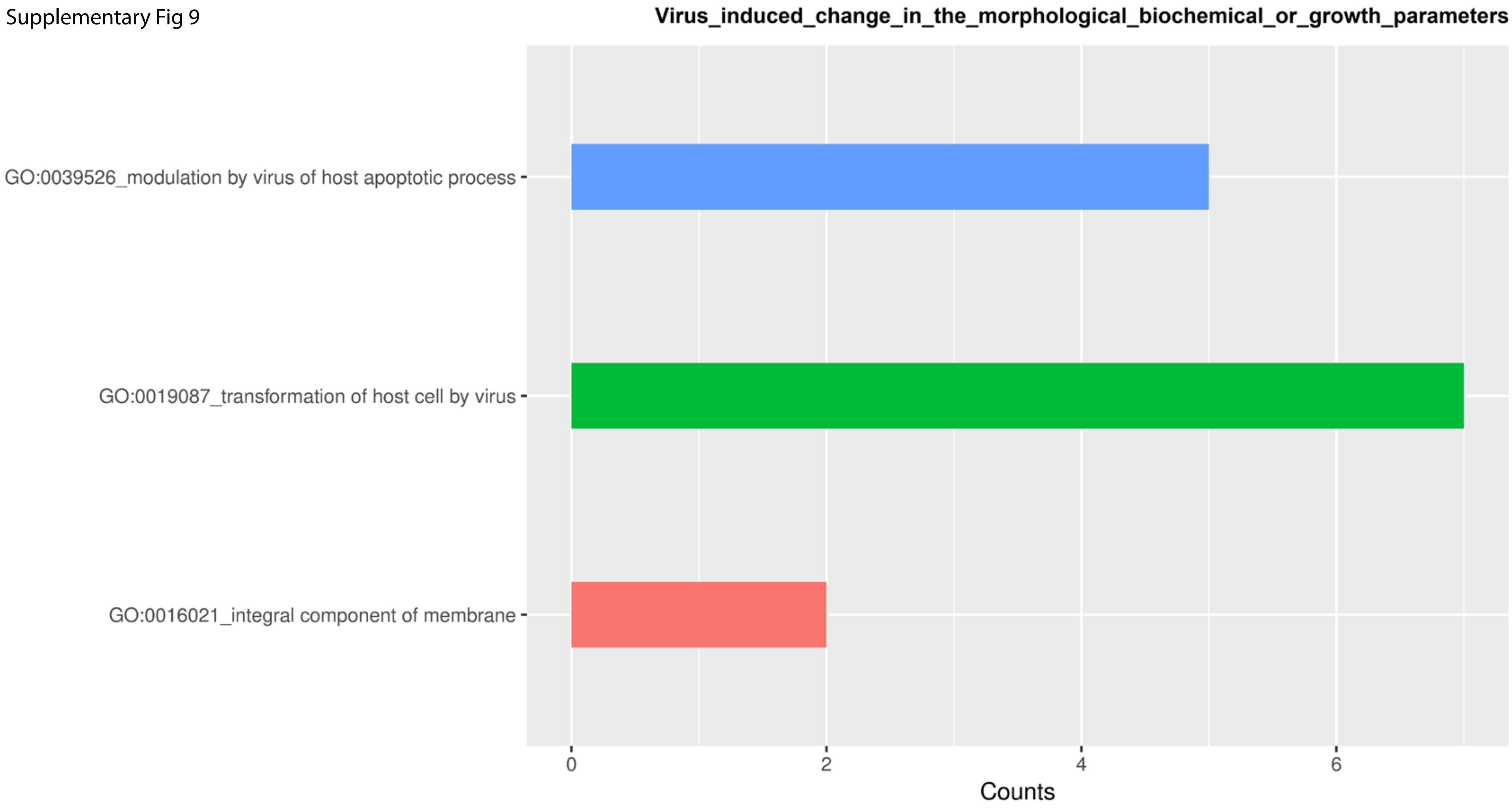

Supplementary Fig 10

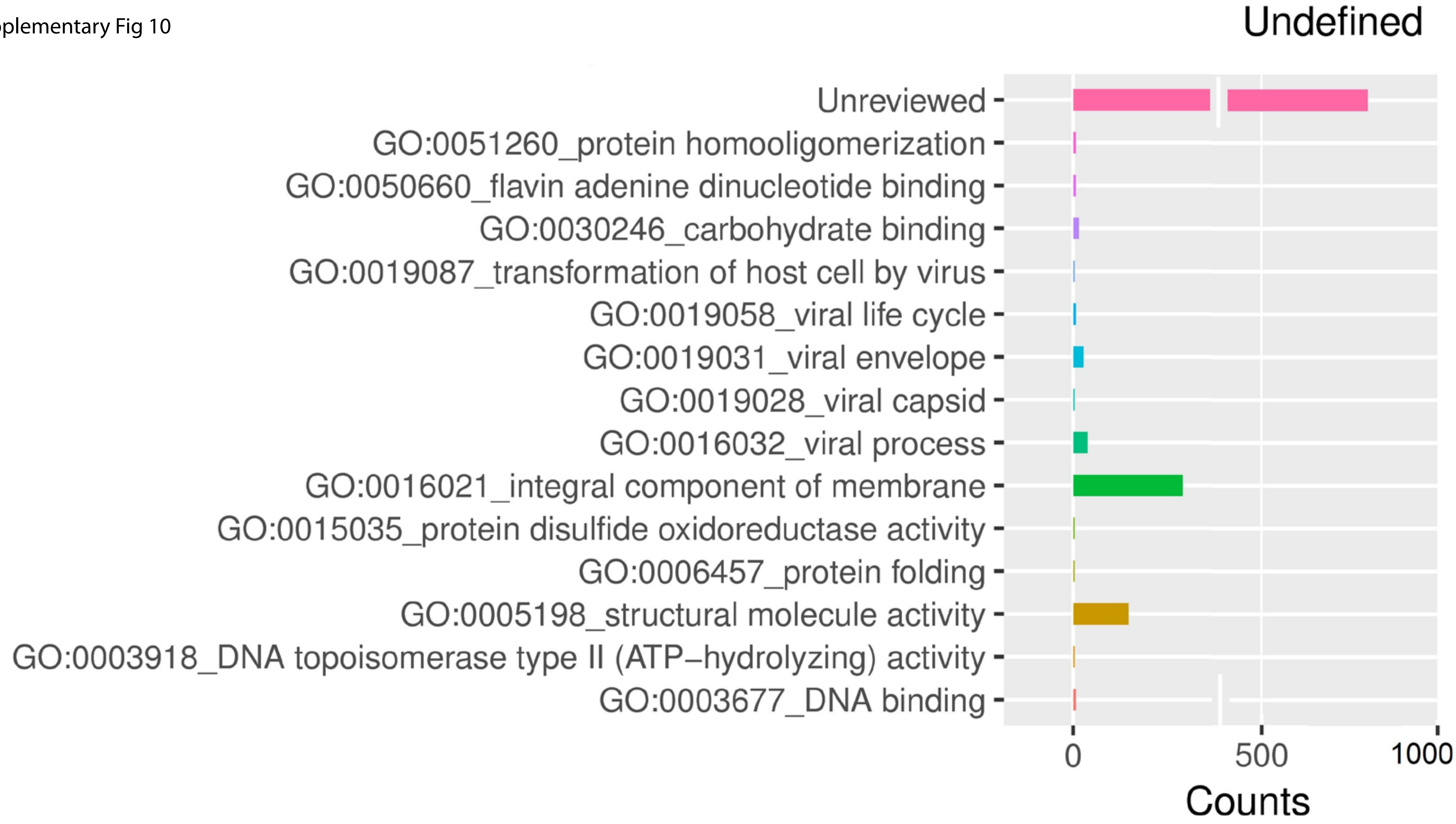

Supplement: Supplementary file 9 — Clustering of viral PrD-containing proteins according to their GO terms [file 41598_2018_27256_MOESM9_ESM.pdf]
